# Clinical application of next-generation sequencing for the management of desmoid tumors: A case report and literature review

**DOI:** 10.1097/MD.0000000000024238

**Published:** 2021-01-08

**Authors:** Jeong Mi Lee, Han Gyeol Kim, So Youn Shin, Seung Hyeun Lee

**Affiliations:** a Department of Medicine, Graduate School, Kyung Hee University; b Division of Pulmonary and Critical Care Medicine, Department of Internal Medicine, Kyung Hee University Medical Center, Kyung Hee University School of Medicine; c Department of Pathology; d Department of Radiology, Kyung Hee University Medical Center, Kyung Hee University School of Medicine, Seoul, South Korea.

**Keywords:** β-catenin, case report, *CTNNB1*, desmoid tumor, lung cancer, next-generation sequencing, thoracotomy

## Abstract

**Rationale:**

Desmoid tumors are rare myofibroblastic neoplasms characterized by local invasiveness and high rates of recurrence, and sometimes mimic local recurrence of previously resected malignancies. Previous studies have suggested that molecular profiling may be useful for the diagnosis of the tumors and risk stratification. However, the clinical utility of next-generation sequencing (NGS) for the management of desmoid tumors has not been established.

**Patient concerns:**

A 42-year-old man visited our clinic for routine follow-up 1 year after left upper lobe lingular segmentectomy for lung adenocarcinoma.

**Diagnoses:**

Chest computed tomography showed a pleural mass adherent to the thoracotomy site. Positron emission tomography revealed mildly increased metabolism with a maximal standardized uptake value of 2.7 within the tumor, suggesting local recurrence of the previous neoplasm. Exploratory thoracotomy and en bloc resection of the tumor revealed spindle cells in a massive collagenous tissue consistent with a desmoid tumor.

**Interventions:**

NGS was performed to confirm the diagnosis and to identify any genetic alterations that might be relevant to the prognosis of this tumor. The tumor harbored an S45F mutation in *CTNNB1*, which has been correlated with a high recurrence rate. Therefore, we performed adjuvant radiotherapy on the resection bed at a dose of 56 Gy.

**Outcomes:**

The patients experienced no postoperative or radiotherapy-related complications. Periodic follow-up examinations using computed tomography were performed every 3 months, and no evidence of recurrence of either tumor was observed during the 38 months after the last surgery.

**Lessons:**

To the best of our knowledge, this is the first case reporting the clinical application of NGS and aggressive treatment based on the genotyping results for the management of a desmoid tumor. Our case highlights the need to consider desmoid tumors among the differential diagnoses when a pleural mass is encountered at a previous thoracotomy site. More importantly, molecular profiling using NGS can be useful for the establishment of a treatment strategy for this tumor, although further investigations are required.

## 1 Introduction

Desmoid tumor, also known as desmoid-type fibromatosis, is a rare mesenchymal soft tissue tumor arising from myofibroblasts.^[1]^ This tumor is characterized by variable clinical behavior and an unpredictable natural course; most tumors gradually grow over time, some are indolent, and spontaneous regression is not uncommon.^[2–5]^ Most desmoid tumors (85–90%) develop sporadically and are associated with somatic mutation of the *CTNNB1* that encodes β-catenin.^[6,7]^ Recent genomic studies have suggested that specific *CTNNB1* genotypes are associated with the risk of recurrence after treatment,^[7–9]^ and the high sensitivity of next-generation sequencing (NGS) facilitates the detection of these genotypes.^[10]^ However, the utility of NGS results for the management of desmoid tumors has not been established.

Here, we report a case of a desmoid tumor mimicking pleural recurrence of a previously resected lung adenocarcinoma. We adopted an aggressive treatment strategy based on the NGS results and obtained a favorable outcome. We comprehensively reviewed previous reports on desmoid tumors that developed after thoracotomy as well as relevant studies on the clinical significance of genomic profiling of these tumors to highlight the possible utility of NGS for patient care and management.

## 2 Case report

**Figure 1:**
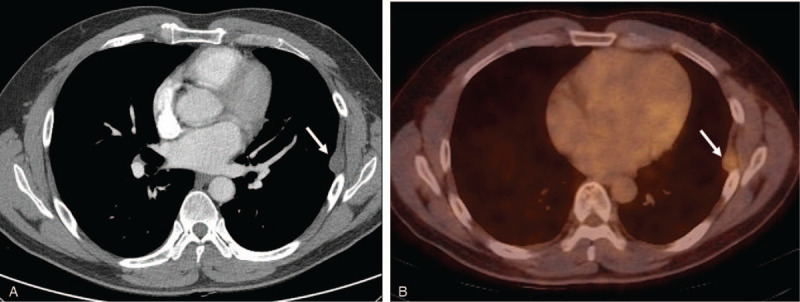
Chest computed tomography (CT) and ^18^F-fluorodeoxyglucose positron emission tomography-computed tomography (PET/CT). (A) Chest CT at a 1-year routine follow-up after left upper lobe lingular segmentectomy for T1bN0M0 lung adenocarcinoma revealed a well-circumscribed, 34-mm diameter solid tumor (arrow) attached to the left pleura, which was the prior thoracotomy site for lung cancer. (B) PET/CT showed mildly increased metabolism with a maximal standardized uptake value of 2.7 within the tumor (arrow).

A 42-year-old man visited our clinic for routine follow-up 1 year after left upper lobe lingular segmentectomy for T1bN0M0 lung adenocarcinoma. He had never smoked and had no medical history. He had no symptoms and physical examination showed no significant findings. Chest computed tomography (CT) showed a newly developed pleural mass on the left hemithorax. The tumor was 34 mm × 25 mm and located in the pleural space (Fig. 1A). Positron emission tomography (PET) revealed mild ^18^F-fluorodeoxyglucose uptake with a maximal standardized uptake value (SUV_max_) of 2.7 within the tumor (Fig. 1B). There was no distant metastasis on PET and brain magnetic resonance imaging. The carcinoembryonic antigen value was 2.38 ng/mL, which was similar to the preoperative value.

**Figure 2:**
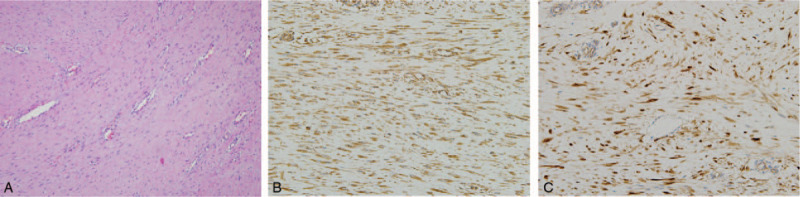
Tumor histopathology. (A) The tumor was composed of proliferating fibroblasts appearing as small bundles of spindle cells in abundant collagenous tissue (hematoxylin and eosin staining). Immunohistochemically, the spindle cells were positive for vimentin (B) and β-catenin (C), which was consistent with desmoid tumor (all × 200).

Given that pleural recurrence of a lung adenocarcinoma was highly suspected, we decided to perform an exploratory thoracotomy. A well-circumscribed ovoid mass was found in the parietal pleura around the fifth intercostal space, which corresponded to the previous thoracotomy site. En bloc resection of the tumor, ribs, and intercostal muscle was performed, followed by chest wall reconstruction. Histopathology showed fibroblastic proliferation appearing as small bundles of spindle cells in abundant fibrous stroma and the spindle cells were positive for vimentin and β-catenin immunohistochemical staining, which was consistent with a desmoid tumor (Fig. 2). Next, we performed NGS to confirm the diagnosis and to identify any genetic alterations that might be relevant for the prognosis of this tumor. A missense mutation S45F (serine to phenylalanine substitution at codon 45) of *CTNNB1* was detected. Previous studies have reported that the presence of *CTNNB1* mutations can be a diagnostic marker for desmoid tumors among spindle-cell tumors, and a specific genotype, S45F mutation, is associated with a high recurrence rate compared to other genotypes.^[7–9,11]^ Therefore, we were able to confirm the patient's diagnosis and predict the aggressive behavior of this tumor. We decided to perform adjuvant radiotherapy to prevent local recurrence of the tumor. A total of 56 Gy was delivered to the resection bed in 28 fractions during the daily course. There were no postoperative or radiotherapy-related complications during the treatment.

The patient underwent periodic follow-up examinations, including laboratory tests and CT every 3 months, and is currently alive without recurrence of lung cancer as well as a desmoid tumor 38 months after the last surgery.

## 3 Discussion

Surgical trauma has been suggested as a risk factor for desmoid tumors, and several cases that develop after previous thoracotomy have been reported.^[12–26]^ Desmoid tumors following thoracotomy for lung cancer often mimic the local recurrence of the previous malignancy and require differential diagnosis. We reviewed the literature and summarized fifteen previous cases and the current case that reported the development of desmoid tumors after thoracotomy in Table 1. The mean age of the patients was 57 years (range, 24–78 years), and cases in males were slightly predominant (56%, 9/16). Ten cases (62.5%) occurred at the site of the previous thoracotomy. Preoperative biopsy was performed in seven cases (43.7%); however, all except one case were non-diagnostic. Prognosis could not be estimated because of the lack of data on recurrence in most of the cases, but the clinical course appeared to be variable. The data suggest that desmoid tumors develop after thoracotomy either at the same or a different site, can occur at any age, and needle biopsy may not be useful for a definitive diagnosis.

**Table 1:** Summary of previous and present cases of desmoid tumors occurring after thoracotomy.

Author, year	Sex/age	Tumor diameter (cm)	PET- SUV_max_	Previous disease for thoracotomy	Occurrence in thoracotomy site	Biopsy results	Treatment	Recurrence (time to recurrence)
Guistra et al, 1979^[12]^	M/42	9	–	Peptic ulcer (vagotomy)	Yes	ND	Resection	Yes (5 years)
Mole et al,1992^[13]^	F/24	25	–	Heart disease	Yes	ND	Resection	Yes (2 years)
Shimizu et al,1999^[14]^	M/74	7.6	–	Lung cancer	Yes	Non-diagnostic	Resection	No
Haoka, et al,2002^[15]^	F/78	8	–	Lung cancer	No	Non-diagnostic	Resection	No
Hashimoto et al, 2002^[16]^	M/56	3	–	Lung cancer	No	ND	Resection	–
Yasuoka et al, 2003^[17]^	F/70	–	–	Lung cancer	-	ND	Resection	No
Tsuboshima et al, 2007^[18]^	F/62	6	–	Lung cancer	Yes	Non-diagnostic	Resection	–
Arimura et al, 2008^[19]^	M/20s	2.5	2.8	Metastasectomy	No	ND	Resection	No
Yoshida et al, 2008^[20]^	F/65	4.5	–	Lung cancer	Yes	Non-diagnostic	Resection	–
Arimura et al, 2010^[21]^	M/60s	3.3	–	Lung cancer	No	ND	Resection	–
Mizutani et al, 2010^[22]^	M/75	8	–	Lung cancer	Yes	Non-diagnostic	Resection	No
Endo et al, 2010^[23]^	F/69	7	–	Lung cancer	No	ND	Resection	–
Zehani-Kassar et al, 2011^[24]^	M/39	11.5	–	Hydatid cyst	Yes	ND	Resection	–
Matsukuma et al, 2012^[25]^	M/76	5.2	–	Lung cancer	Yes	Desmoid tumor	Resection	No
Mori et al, 2014^[26]^	F/62	7.4	2.7	Lung cancer	Yes	Non-diagnostic	Resection	No
The present case, 2020	M/42	3.4	2.7	Lung cancer	Yes	ND	Resection plus radiotherapy	No

As desmoid tumors often mimic the local recurrence of a previous malignancy, PET can be a useful tool for differential diagnosis. Only three cases, including the present case, have described PET data and reported that tumors exhibit increased metabolism (2.7–2.8 of SUV_max_).^[19,26]^ Kasper et al analyzed the PET results of 16 desmoid tumors and demonstrated that SUV_max_ was variable, ranging from 1.0 to 8.1 with a median of 4.1.^[27]^ These data indicate that PET may not be useful for the discrimination of desmoid tumors from the recurrence of previously resected malignancies.

Studies have shown that dysregulated wound healing is involved in the pathogenesis of desmoid tumor-like fibroblastic lesions.^[28]^ In particular, the sporadic type of desmoid tumor is associated with *CTNNB1* mutations, which dysregulate the β-catenin level and induce nuclear accumulation of β-catenin.^[29,30]^ Recent molecular studies have demonstrated that mutational analysis for *CTNNB1* is clinically significant for the diagnosis of desmoid tumors. Le Guellec et al performed genomic sequencing using 260 desmoid tumors and 191 desmoid-like spindle cell lesions.^[11]^ They demonstrated that 88% of the desmoid tumors showed exclusively *CTNNB1* mutations, suggesting that detection of these mutations could be useful for discriminating desmoid tumors from other spindle cell tumors.^[11]^ A subsequent study using various molecular profiling methods, including whole-exome sequencing, demonstrated that the genetic alterations in *CTNNB1* were almost universal in sporadic desmoid tumors.^[31]^

Despite complete resection, desmoid tumors have a high rate of recurrence, and the contribution of incomplete resection to local recurrence rate remains debatable. A meta-analysis of 16 retrospective studies revealed that patients with microscopically positive resection margins had a significantly higher recurrence risk.^[32]^ In contrast, another study reported high recurrence rates (up to 38%) even in tumors that were aggressively treated with resection with widely negative margins.^[33]^ Although the anatomic site of disease, size, gender, and age have been suggested as factors associated with the recurrence rate, the significance of each variable has been inconsistent across studies.^[3,34,35]^ Therefore, finding predictive factors for recurrence is essential for the proper management of desmoid tumors.

Interestingly, molecular subtypes of *CTNNB1* or specific gene expression signatures are emerging as biomarkers for recurrence risk.^[6–9,36–38]^ We have summarized previous studies on the putative molecular biomarkers for desmoid tumors in Table 2. An early study showed that overexpression of β-catenin and p53 was related to a high local recurrence rate.^[36]^ Other studies have suggested that the presence of *CTNNB1* mutations, midkine expression, or certain molecular signatures are associated with local recurrence.^[7,9,37]^ Of note, several studies have demonstrated that a specific mutation of *CTNNB1*, S45F, is a reliable molecular predictor of local recurrence.^[6,8,38]^ Lazar et al first demonstrated that S45F was associated with high recurrence rate (23% of 5-year recurrence-free survival in S45F-mutant tumors compared to 57% in S41A-mutant and 65% in wild-type tumors).^[6]^ Colombo et al and van Broekhoven et al confirmed the predictive value of the genotype in separate studies, although one study found no association between the genetic alterations and recurrence rate.^[8,38,39]^ A subsequent report indicated that NGS has high sensitivity (92.3%) and specificity (100%) for the detection of the *CTNNB1* mutations in desmoid tumor-like spindle cell lesions, suggesting that the assay may be clinically useful for the detecting mutations.^[10]^ Given that we detected S45F mutation following NGS, we opted to treat the patient using adjuvant radiotherapy even though the tumor was radically resected. To the best of our knowledge, this is the first report of clinical decision- making and aggressive treatment based on NGS genotyping for the management of desmoid tumors. Although repeated resection, radiotherapy, and systemic treatment are used either alone or in combination, the optimal management of patients with desmoid tumors is yet to be determined due to its rarity and unpredictable behavior.^[1]^ Moreover, the utility of post-operative radiotherapy remains inconclusive. The National Comprehensive Cancer Network guideline suggests that postoperative radiotherapy should be considered after R2 resection or in the setting of disease progression or recurrence, with or without surgery.^[40]^ The role of adjuvant radiotherapy and optimal treatment strategy, especially for tumors that are predicted to be aggressive based on genotyping, should be the subject of future studies.

**Table 2:** Summary of literature review on molecular biomarkers for desmoid tumors.

Author, year	Patients number	Method	Factors associated with higher local recurrence
Gebert et al, 2007^[36]^	38	Tissue microarray and IHC	β-catenin and p53 overexpression
Lazar et al, 2008^[6]^	160	Direct sequencing for *CTNNB1* mutations	S45F mutation of *CTNNB1* and β-catenin underexpression
Dômont et al, 2010^∗^^[7]^	101	Direct sequencing for *CTNNB1* mutations	Positive *CTNNB1* mutations
Colombo et al, 2011^[37]^	14	Tissue microarray	Enhanced midkine expression
Mullen et al, 2013^[39]^	145	Single-base extension genotyping for *CTNNB1*	No factors found
Colombo et al, 2013^[8]^	179	Direct sequencing for *CTNNB1* mutations	S45F mutation of *CTNNB1*
van Broekhoven et al, 2015^[38]^	101	Direct sequencing for *CTNNB1* mutations	S45F mutation of *CTNNB1*and young age
Salas et al, 2015^[9]^	115	cDNA microarray	36-gene molecular signature

## 4 Conclusion

Diagnosis of desmoid tumors is usually incidental and challenging, particularly in cases with no clinical symptoms. Although tumors rarely metastasize distantly, they are often locally invasive. Occasionally, desmoid tumors develop at the site of previous surgical resection mimicking local recurrence of a previous malignancy. Here, we describe a rare case of a desmoid tumor arising at the antecedent thoracotomy site. As the molecular profiling demonstrated that the tumor harbored the *CTNNB1* S45F mutation which is associated with an aggressive phenotype, we performed adjuvant radiotherapy even after radical resection.

Our case highlights the potential role of NGS as a supplementary tool for diagnosis and risk stratification, although other factors influencing clinical outcome and the optimal therapeutic strategy remain to be established. In addition, desmoid tumors should be included in differential diagnoses when clinicians encounter a chest wall mass in a patient who has previously undergone thoracotomy.

## Acknowledgments

The authors thank the patient and his family for permission to publish this case report.

## Author contributions

**Conceptualization:** Seung Hyeun Lee.

**Data curation:** So Youn Shin, Seung Hyeun Lee.

**Formal analysis:** Jeong Mi Lee, Han Gyeol Kim, So Youn Shin, Seung Hyeun Lee.

**Funding acquisition:** Seung Hyeun Lee.

**Investigation:** Han Gyeol Kim, Seung Hyeun Lee.

**Methodology:** Han Gyeol Kim, Seung Hyeun Lee.

**Project administration:** Seung Hyeun Lee.

**Supervision:** So Youn Shin, Seung Hyeun Lee.

**Validation:** Jeong Mi Lee, So Youn Shin, Seung Hyeun Lee.

**Visualization:** Jeong Mi Lee, Seung Hyeun Lee.

**Writing – original draft:** Jeong Mi Lee, Seung Hyeun Lee.

**Writing – review & editing:** Jeong Mi Lee, Seung Hyeun Lee.
